# ITD assembler: an algorithm for internal tandem duplication discovery from short-read sequencing data

**DOI:** 10.1186/s12859-016-1031-8

**Published:** 2016-04-27

**Authors:** Navin Rustagi, Oliver A Hampton, Jie Li, Liu Xi, Richard A. Gibbs, Sharon E. Plon, Marek Kimmel, David A. Wheeler

**Affiliations:** Human Genome Sequencing Center, Baylor College of Medicine, Houston, TX USA; Department of Statistics, Rice University, Houston, TX USA; Department of Molecular and Human Genetics, Baylor College of Medicine, Houston, TX USA; Department of Dermatology, Xiangya Hospital, Central South University, Hunan, China; Department of Pediatrics/Hematology-Oncology, Texas Children’s Hospital, Houston, TX USA

**Keywords:** Tandem duplication, De Bruijn graphs, Assembly, FLT3, Data mining, Cancer genetics, AML, Clustering, Somatic mutations

## Abstract

**Background:**

Detection of tandem duplication within coding exons, referred to as internal tandem duplication (ITD), remains challenging due to inefficiencies in alignment of ITD-containing reads to the reference genome. There is a critical need to develop efficient methods to recover these important mutational events.

**Results:**

In this paper we introduce ITD Assembler, a novel approach that rapidly evaluates all unmapped and partially mapped reads from whole exome NGS data using a De Bruijn graphs approach to select reads that harbor cycles of appropriate length, followed by assembly using overlap-layout-consensus. We tested ITD Assembler on The Cancer Genome Atlas AML dataset as a truth set. ITD Assembler identified the highest percentage of reported FLT3-ITDs when compared to other ITD detection algorithms, and discovered additional ITDs in *FLT3*, *KIT*, *CEBPA, WT1* and other genes. Evidence of polymorphic ITDs in 54 genes were also found. Novel ITDs were validated by analyzing the corresponding RNA sequencing data.

**Conclusions:**

ITD Assembler is a very sensitive tool which can detect partial, large and complex tandem duplications. This study highlights the need to more effectively look for ITD’s in other cancers and Mendelian diseases.

**Electronic supplementary material:**

The online version of this article (doi:10.1186/s12859-016-1031-8) contains supplementary material, which is available to authorized users.

## Background

Somatic internal tandem duplication (ITD) mutations are an important oncogenic driver in Acute Myeloid Leukemia (AML), where an ITD within the juxta-membrane domain of FLT3 confers poor prognosis [1]. Both the length and the somatic allelic ratios have been shown to affect patient outcomes [2]. FLT3-ITD's, ranging in size from 15 to 300 bp, are found in 20–30 % of AML patients and are associated with increased risk of disease relapse and decreased overall survival [3, 4]. Patients harboring FLT3-ITDs are treated more aggressively. Sensitive detection of FLT3-ITD mutations in AML patients is therefore clinically important.

Detection of insertions in short read NGS data is a challenge because insert-containing reads often fail to align to the reference genome using BWA [5], causing poor ascertainment of insertions in 15–80 bp range. Reads that contain or span the junction of tandem duplications will either be marked as unmapped or only partially aligned (referred to as “softclipping” in Sequence Alignment/Map format [6]). Detection of ITDs using *de novo* assembly methods may partially overcome the limitations of alignment-based algorithms; however, even *de novo* sequence assembly approaches may fail to assemble duplicate regions of genomes [7–9]. Construction of De Bruijn graphs, by kmer-based assembly methods, results in loops in the De Bruijn graph, potentially causing duplicated regions to collapse and making it difficult to accurately represent complex repetitive sequence structures. Alternatively, overlap-layout-consensus (OLC) assembly methods accurately assemble duplicated regions, but are not efficiently scalable to datasets typical of current next generation sequencing technologies.

To overcome these limitations, we developed a two-step assembly approach, ITD Assembler, which relies on the strengths of both assembly strategies to discover duplicated inserts on a length scale consistent with known ITDs. In this approach, all unmapped and softclipped reads, which partially align to the genome, are subjected to a kmer frequency spectrum analysis and De Bruijn graph assembly process. From this analysis we accurately identify groups of candidate reads that span a tandem duplication. Reads containing these ITD signatures are then assembled into contigs using an OLC algorithm and subsequently mapped back to the reference genome.

ITD Assembler takes a bam file as input, and outputs an annotated bed file. It performs a kmer frequency analysis to infer the putative read sets containing duplications and includes an optional De Bruijn graph construction-based resource optimization module, easily customized for user specific computing constraints and mutational allele fraction requirements. It can detect tandem duplications with an upper bound that is 10 bp less than read length, lower bound of length 10 bp, and scales well with larger length reads. Detection sensitivity is a function of user requirements on runtime and memory. Read assembly is accomplished with the OLC based assembly algorithm Phrap [10], which is run with parameters agnostic to read fragment information and quality, and outputs an ace file for each of the contigs. A post processing annotation pipeline performs contig alignment to the reference and filters alignments capturing internal tandem duplication in the human reference sequence.

There has been some past work on using assembly based approaches for detecting insertions, which also apply to detecting tandem duplications [11–13]. In the tool MindTheGap [11], a De Bruijn graph based approach is used to infer insertion events followed by a kmer based De Novo assembly of putative insertion sites. While we also perform De Novo assembly of contigs at the last stage, our pipeline only uses De Bruijn graphs to identify reads which could harbor duplications. The assembly of tandem duplications is always carried out using Phrap, thereby overcoming any limitations of De Bruijn graph based methods in resolving complex tandem duplications.

In the tool ANISE [12], an OLC method is used for assembling insertions, but the assembly process is guided only by unmapped reads which have their mates mapped. While ITD Assembler also uses alignment information indirectly by selecting unmapped reads, inference of breakpoints is carried out post assembly thereby reducing reference bias in detecting tandem duplications. The FermiKit algorithm [13] also does indel discovery post assembly, but is focused on short insertion events. While all the algorithms presented above overlap with some aspects of assembly-based insertion discovery, the design of the ITD Assembler algorithm is novel in the context of finding tandem duplications, and is specifically tuned to provide high sensitivity in their detection.

Algorithms relying on an unusual presence of softclipped reads or reads with a mapped mate may miss detecting tandem duplications captured with low coverage or tandem duplications with novel sequences. Since ITD Assembler makes use of all unmapped reads along with softclipped reads, it has high detection sensitivity for such variants.

ITD Assembler was applied to 314 AML patient samples (including a leukemic and normal tissue pair) from 157 AML patients sequenced by whole exome capture to an average of 165X coverage, with corresponding RNA sequencing data, all within the TCGA program [14].

## Methods

### Algorithm overview

**Fig. 1:**
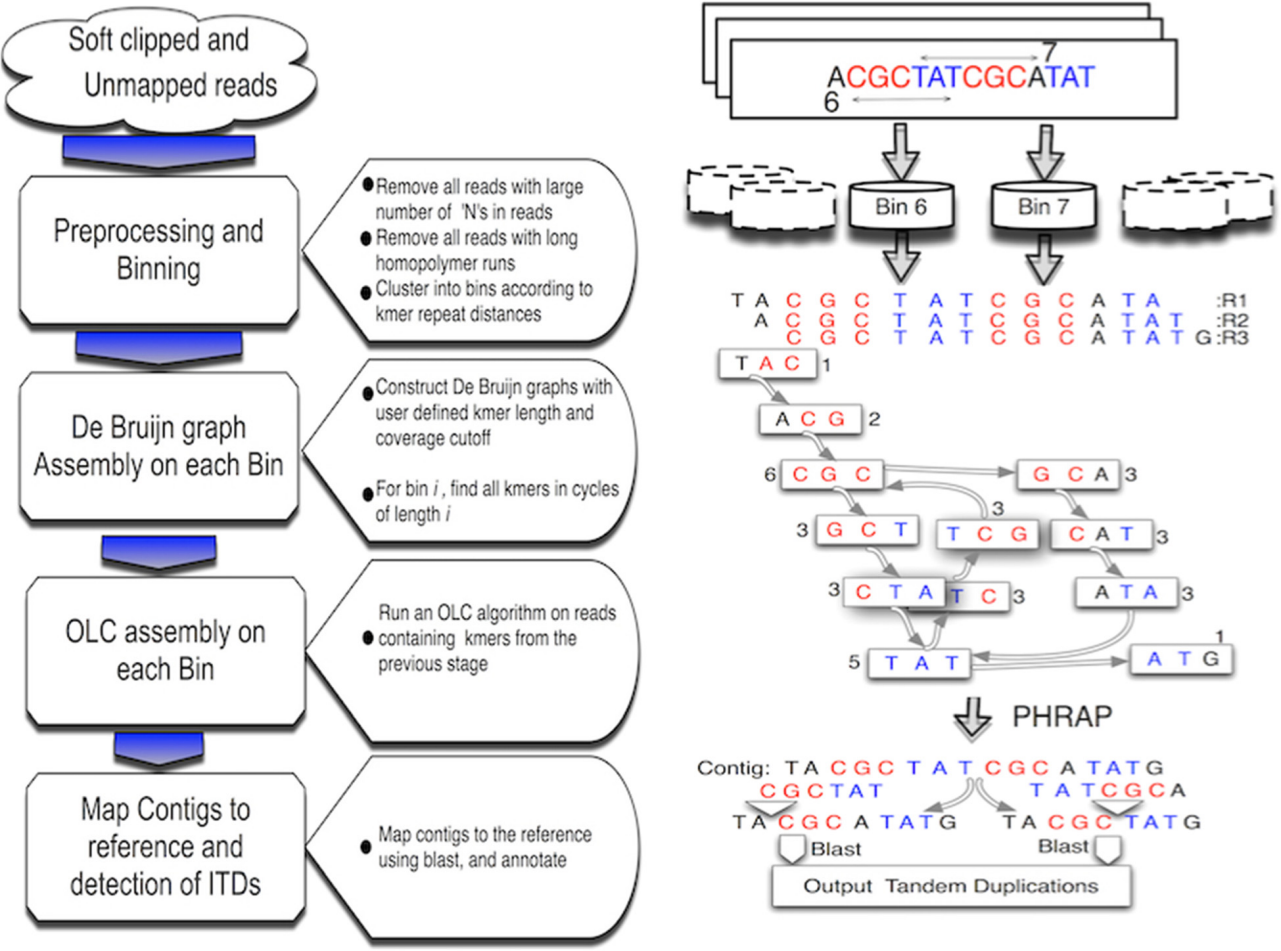
ITD Assembler workflow schema. The major ITD Assembler processing steps are depicted with transitional blue arrows decreasing in size, representing a serial reduction in the number of reads at each step. To the left of the ITD Assembler workflow is an example read set containing two repeated kmers, one 6 bases and another 7 bases apart, which are placed into bins 6 and 7. De Bruijn graph construction is performed on these three example reads using, for illustration purposes, kmer length 3. The integer values near each vertex are the coverage of each vertex in the graph. There are two cycles of length 6 and 7 in this graph representing the two independent ITDs. OLC assembly is performed on those read sets from bins 6 and 7, with resulting contigs being annotated and ITDs reported

Beginning with the entire set of unmapped and soft-clipped reads from a binary alignment/map (BAM) file, ITD Assembler progressively filters reads through a series of steps to produce groups of reads for OLC assembly using Phrap [10] (Fig. 1). In the first stage of the algorithm, all reads not containing the signature of a tandem duplication are eliminated from the pipeline using a stringent filtering criteria and kmer frequency analysis. The algorithm then performs an overlap clustering of the remaining reads, where each cluster contains reads of a particular length tandem duplication signature (these clusters are referred to as bins). A De Bruijn graph is constructed from reads in each bin, with kmers of those reads constituting the vertices of that graph. A fast matrix exponentiation method evaluates the adjacency matrix of the De Bruijn graph to find all cycles of the representative bin length supported by kmer coverage above a user defined cutoff. Only reads that contain kmers that participate in a cycle are included from that bin for further analysis. The reads remaining at the end of this stage are assembled using Phrap. Contigs from Phrap are then compared to the human reference using BLAST [15] to annotate their origin relative to coding exons, and the proportion of reads supporting the ITD, which are reported as a proportion of the total coverage over the target (i.e., the allele fraction of the ITD). To this point, ITD Assembler has identified tandem duplications over the range of 15–80 bp. Analysis of the BLAST alignments separates the tandem duplications representing insertions from those that do not, and annotates those representing insertions as coding (ITD) or intronic. An ancillary component to the post processing pipeline compares the contigs to RNA sequencing data, if available, to verify expression of the mutation. For exome sequencing experiments, contigs that do not map to a target interval in the user defined capture region are removed from further analysis.

### Preprocessing and binning step: input parameters = p_kmer,(r_min, r_max)

Notations are as follows: A kmer of a string of ***S*** of size ***k***, is defined as any contiguous substring of k bases. Let ***N***_*k*_**(*S*)** be defined as the set of all kmers of a string S. Let ***C***_***S***_**(X)** be the number of times base **X** occurs in the string ***S***. Let **|*S*|** be the length of the string ***S***. For a De Bruijn graph ***G***, let ***A***_***G***_ be the adjacency matrix of this graph and for any matrix ***A*** let ***A***^***i***^ be the ***i***^**th**^ power of ***A***.

All the unmapped reads and soft-clipped reads with an unaligned head or tail segment of at least 4 base pairs are extracted from the bam file using SAMtools [5] and BamTools [16]. The subsequent steps of this stage of the pipeline are as follows:

- Identify all unmapped and soft-clipped reads with soft-clipped regions ≥4 bp.
- Identify and filter unmapped reads ***S*** where ***C***_***S***_**(‘*N*’)** > **50**.
- Identify and filter unmapped reads ***S*** with homopolymer runs of 15 or more.
- Identify and filter unmapped reads ***S*** which do not contain a duplication sequence signature of defined length *p_kmer*, i.e. if ***N***_***p_kmer***_**(*S*) = |*S*|-**(*p_kmer*) + 1. Remaining unmapped reads are annotated with unique distances between the starts of the two duplicated kmer patterns.
- Binning: all reads annotated with distance ***i*** in the previous step are clustered into bin ***i***. (see Fig. 1). In case of multiple duplication lengths, the same read will go to all bins with a representative duplication length.

The parameter *p_kmer* is the partial tandem duplication parameter. Once initialized, ITD Assembler will not be sensitive to any partial duplication with less than *p_kmer* length. The variables *r_min* and *r_max* are user-defined variables that specify the range of ITD lengths for which bins are created. This range is upper bounded by **|S|**-*p_kmer*, where **|S|** is the read length. This algorithm cannot find tandem duplications outside the range defined by [*r_min*, *r_max*].

The treatment of soft-clipped reads is similar to the unmapped reads, with *p_kmer* assigned the value of the length of the soft clipped region. For unmapped reads, a kmer frequency analysis is done for all kmers, whereas in the soft clipped reads we search for duplications of the soft clipped region only.

### De Bruijn graph assembly: input parameters = d_kmer, cov_cutoff_min, cov_cutoff_max

In this step, the algorithm iterates over each length in the user defined range, and constructs a De Bruijn graph using the reads in that bin with kmer size *d_kmer*. The value of *d_kmer* cannot be less than partial tandem duplication parameter *p_kmer* (defined in the previous section), but making *d_kmer* larger than *p_kmer*, one will only be able to detect partial tandem duplications of length *d_kmer* and above, by default *d_kmer* = *p_kmer*. Let ***G***_***i***_ be the subgraph of the De Bruijn graph constructed on bin ***i*** with only vertices having coverage between *cov_cutoff_min* and *cov_cutoff_max* included in ***G***_***i***_. The algorithm uses the standard exponential by squaring method to compute ***A***^***i***^_***Gi***_. Reads devoid of kmers that are part of ***i***-cycle in ***A***_***Gi***_ are excluded from further analysis.

### OLC assembly and annotation

ITD Assembler then iterates through all the bins, taking kmer read subsets that formed De Bruijn graph cycles and assembles them into contigs using the OLC assembler Phrap. The computational overhead of Phrap is within reasonable resource bounds for a typical super-computing environment. The Phrap produced contigs are then passed through an annotation pipeline that derives genome location, affected genes and mutational allelic fraction (i.e. the number of reads supporting the ITD). In the annotation pipeline, each contig that has a duplicated kmer of size *d_kmer* (see previous subsection) is selected for further analysis and the region between the repeated kmer is deleted from the contig. It is then aligned to the reference genome using BLAST [15]. Local alignments with high mapping specificity are annotated with the targeted capture bed file (in the case of exome capture sequencing) to assign coordinates and gene names to the contigs. Phrap is run with default parameters and all quality values are ignored in this version of the algorithm.

### Test case: FLT3 detection in AML

ITD Assembler was applied to 314 AML patient samples (representing matched leukemic and normal tissue) from 157 AML patients sequenced as part of the TCGA program [14]. Whole exome sequencing achieved 165X-fold average coverage. The TCGA AML study reported 22 patients harboring somatic FLT3-ITD mutations, providing a positive control set for comparison [14]. Assessment of sensitivity was performed by cross-validation of FLT3-ITD detection employing the ITD Assembler tool, the orthogonal tandem duplication detection tools Pindel, Genomon ITDetector and published TCGA FLT3-ITDs reported from RNA-seq data using Barnacle v0.1 [14]. TCGA AML samples exhibiting evidence of somatic FLT3-ITD mutation from any of the four methods are detailed in Table 1.

**Table 1:** Somatic FLT3-ITD detection in TCGA data

TCGA ID	WES Avg Cov	Read length	TCGA^a^ ITD (length)	ITD Asm (length)	ITD Asm (Mut af)	Pindel^b^ (length)	Pindel (Mut af)	Genomon^c^ (length)
2853	182.3	100	18	18	0.011			
2840	124.1	100	18	18	0.011			
2877	225.2	75	18	18	0.232			18
2880	180.4	75	21	21	0.254			21
2918	76.5	75	21	21	0.113			21
2942	212.7	75	24	24	0.159			24
2875	229.9	75	30	30	0.187			30
2836	217.7	75	33	33	0.054			
2879	186.9	75	33	33	0.355			33
2922	172.8	75	33	33	0.262			33
2925	141.5	75	42	42	0.291	42	0.024	42
2930	142.1	100	42					42
2895	151.4	75	45	45	0.201	45	0.059	45
2812	206.3	75	51	51	0.412	51	0.109	
2869	198.8	100	54	54	0.017			54
2830	267.7	75	69	69	0.038	56	0.025	42
2921	119.3	100	24					24
2871	210.2	75	63					
2913	100.5	75	66			66	0.085	66
2931	71.1	75	75			70	0.316	
2844	223.5	75	87			87	0.156	
2825	98.2	100	102			102	0.023	
2809	113.6	100		30	0.019			
2949	174.1	75		39	0.021			
2915	75.2	75		51	0.058	49	0.024	51
2895	151.4	75		51	0.228	50	0.051	
2934	149.9	75		57	0.058	56	0.071	57
2823	212.0	75		57	0.137	57	0.062	
2833	206.1	75		75	0.004			
2862	207.8	75				69	0.200	69
2923	197.1	75				74	0.025	74
2918	76.48	75				88	0.097	88
2919	189.8	75				93	0.022	93
2959	164.9	75				118	0.049	
2896	146.6	75				153	0.084	153
2921	119.3	100						57
Total			22	22		18		22

All lengths are in base pairs

^a^reference 1, TCGA AML marker paper used as reference set for algorithmic discovery

^b^reference 23, Ye et al. [18]

^c^reference 24, Chiba et al. [17]

## Results and discussion

**I**TD Assembler was evaluated on The Cancer Genome Atlas (TCGA) 157 acute myeloid leukemia (AML) patient cohort consisting of both leukemic and normal whole exome sequencing experiments, for which 22 FLT3-ITD mutations were reported but without supporting allele fraction information. We compared results of ITD Assembler to the TCGA report and other available methods to assess its sensitivity (Table 1). The TCGA project consisted of whole exome sequence in which most patients had 75 bp reads. The range of detection for ITD Assembler for 75 bp reads is 15–60 bases, and for 100 bp reads is 15–80 bp.

As shown in Table 1, ITD Assembler detects 15 of 22 of the reported FLT3 ITDs. Of the 7 missed, 5 were longer than the read length allowed (>60 bp in a 75 bp read). This was superior to the recently published Genomon [17], the next best detector, which found 14 of the 22 reported, and Pindel [18], which found 8 of 22. Pindel version 0.2.4q was run on this dataset with default parameters, whereas the data release version was used for comparisons with Genomon.

Comparing ITD Assembler and Pindel (the only two tools that report mutant allele fractions), Pindel found only half as many mutant alleles as ITD Assembler. Furthermore, in the cases where ITD Assembler and Pindel reported the same ITDs, higher mutational allele fractions were found by ITD Assembler.

All three detectors found unique ITDs in FLT3. Genomon-ITDetector(Genomon) discovered 8 and Pindel found 10; however, 6 of the 10 found by Pindel and 5 of 8 found by Genomon were out of the size range that ITD Assembler could discover. ITD Assembler identified a single patient exhibiting two distinct somatic FLT3-ITDs with lengths 51 bp and 45 bp and similar allele fractions of 0.228 and 0.201 respectively only one of which, the 45 bp ITD, was reported by TCGA. Pindel also found the additional ITD in this patient. There is evidence that FLT3-ITD bi-allelic mutations in AML confer resistance toward *PTK* inhibitors and cytotoxic agents [19], and could be clinically relevant.

For ITDs detected by both ITD Assembler and Pindel, the mutant allele fractions (Mut af, Table 1) were always higher in ITD Assembler output than with Pindel. Genomon did not report allele fractions, an important disadvantage. Under the sequencing constraints of Illumina paired-end reads, often the paired reads overlap and therefore both reads of the pair may harbor the ITD and both may fail to map. Thus the improvement in sensitivity in ITD Assembler comes, in part, from the fact that we use all unmapped reads. Both Pindel and Genomon use unmapped reads only if the mate pair maps.

ITD Assembler found somatic ITDs in several other cancer genes within the TCGA AML cohort. One patient harbored a KIT-ITD involving exon 11 and 12 of its membrane domain. KIT-ITD mutation has a reported occurrence of approximately 7 % in AML, and was previously shown to be an activating oncogenic mutation [20]. Consistent with the report of Genomon, two somatic WT1-ITDs, were identified. Overall, ITD Assembler discovered a total of 1322 somatic ITDs, in 469 genes, in the TCGA AML cohort. We also found 243 germline ITD mutations in 54 genes.

Among these we identified a number of germline ITD involving cancer driver genes, the most notable being the AML target gene *CEBPA*, a transcription factor involved in myeloid differentiation and known to be lost in approximately 50 % of AML cases [21]. *ACSF3* is the gene with the highest Minor Allele Frequency (MAF = 0.16) of germline ITD mutation. ITD’s were also identified in *AURKA. AURKA* is aberrantly expressed in chemotherapy-resistant *CD34(+)/CD38(−)* AML cells and thus may be a promising molecular target in AML [22].

We validated ITDs in two ways (Additional file 1). As a visual check we verified that the target of the duplication was present in at least two copies in the assembled contig and that the duplicated sequence was found within an exon (see Additional file 1: Figure S2) Second we sought support for ITD expression using TCGA RNAseq data, which was available for 128 of 157 AML patients. For each patient RNA sequencing reads were aligned using BLAST to the patient’s assembled contigs containing putative ITDs. RNA sequencing reads that aligned with greater than 97 % identity, crossed ITD junctions and exhibited adequate coverage values were considered validating evidence for ITD mutation. Of the transcripts that were expressed with at least 10 reads, ITDs were validated in 74.3 % of somatic and 70.3 % of germline events.

We have shown a novel method for discovery of ITDs in DNA sequencing data, which is effective in both tumor and normal genomes. ITD Assembler attains a higher sensitivity than existing approaches, but is currently limited in length of ITD to the length of the reads provided. We have two strategies to overcome this limitation. First as Illumina reads increase in length our detection range will automatically increase. Second we can potentially merge mate pairs before initiating our kmer analysis to artificially increase read lengths. Both strategies are being investigated.

### Computational complexity and optimization

ITD Assembler is implemented in Haskell, with some intermediate parts in C and Python. It can run on a single core. While no inbuilt parallelization has been implemented in it, the bins can be run on separate cores in the Assembly stage.

For a read S, the binning and soft clip extraction make extensive use of a fast sorting routine on kmer spectrum of S to filter out reads without any kmer repeats in them in expected O(|S|log|S|) time. The size of the adjacency matrix for each bin scales quadratically with the number of unique kmers of length *d_kmer* within the bounds set by the *cov_cutoff* parameters. Increasing the value of *d_kmer* does reduce the number of unique kmers, however there is a length at which the number of unique kmers is subject to diminishing results, and estimating an optimized length *d_kmer* parameter in this context is open to further investigation. Also, while increasing the value of *d_kmer* may be beneficial in terms of runtime and resource consumption, it reduces the partial detection sensitivity to length *d_kmer.*

The matrix multiplication module from the GSL-CBLAS library is used to compute cycles from the adjacency matrix. Therefore the De Bruijn graph filtering stage takes time *O(kn*^*3*^*)* where *n* is the number of unique kmers in the bin and *k* is the bin number. Low complexity regions increase the noise in detecting true signals of detection sensitivity and is open to further investigations. The Phrap assembly depends on underlying genomic complexity, where the time complexity is highest for assembling low complexity regions.

To accommodate high detection sensitivity and concurrent runs of 157 tumor/normal pairs in the local compute environment with maximum of 45G of memory per node, it was necessary to do away with the increased memory footprint of the De Bruijn graph construction at the cost of potentially increasing the run time. We refer to the version that does not use De Bruijn graph assembly as ITD Assembler_*light.* In this version all other steps are the same as is described in section 2, except that Phrap is run on reads in each bin without the De Bruijn graph based read filtering. The biggest performance bottleneck in the ITD Assembler pipeline is the Phrap assembly of short reads.

For values *p_kmer* = 10, *r_min* = 15 and *r_max* = 61, ITD Assember_*light* completes processing in approximately 5 h for samples with read length 75 bp and approximately 1.5 days for read length 100 bp on nodes based on intel Xeon E5520 and AMD Opteron architecture for most of the samples. The binning step takes approximately 1.5 h. for length 75 base pair reads and approximately 3 h for length 100 bp reads. For a sample that took 9 days to complete using ITD_Assembler_light, ITD_Assembler is able to finish the whole pipeline in 16 h and 15G of memory with parameters *p_kmer* = 15, *d_kmer* = 15, *cov_cutoff_min* = 15, *cov_cutoff_max* = 500, *r_min* = 15 and *r_max* = 85. If the computation on each bin is parallelized across 60 cores, the pipeline can finish in 5 h given the same parameters.

### Application of ITD Assembler on FLT3 detection sensitivity

ITD assembler is executed on the 22 samples with FLT3 somatic tandem duplications with parameters p_kmer = 15, d_kmer = 15, cov_cutoff_min = 30,,cov_cutoff_max = 200,r_min = 15 and r_max = 85 . 15 out of 22 samples were detected to have an ITD with these parameters (see Additional file 1: Table S1). The minimum kmer coverage for all the kmers making the tandem duplication was above 30 for these 15 samples. A value of cov_cutoff_min = 30 translates to detecting any ITD with alternate allele fractions greater than 0.2, which is a lower bound for high quality assurance in the Genomon paper as well [15]. ID 2830 is missing from this run of ITDAssembler but is detected by Pindel and Genomon with length different from the TCGA length. It is to be noted that detecting tandem duplications in id 2830 and 2833 are beyond the intended detection range of ITD Assembler/Assembler_light but are chance discoveries from ITD Assembler_light. Both of these ITD’s have supporting RNAseq data. Decreasing the cov_cutoff_min = 15 detects two more tandem duplications (see Additional file 1: Table S1). Of all the missing tandem duplications at the end of this run, there is only one which is detected by both Pindel and Genomon, i.e. ID 2934. This ITD has one constituent kmer with kmer coverage of 6 which is below the threshold set up cov_cutoff_min. Another run on the missing samples with lower cov_cutoff_min parameters failed to add to the existing set of ITD’s. Changing the d_kmer value to 10 and cov_cutoff_min value to 5 made ITDAssembler run for more than two days and was killed at that stage.

We observe that while running the De Bruijn graph optimization makes hard to assemble bins tractable by removing reads which consist entirely of kmers which are highly repetitive, and reads which have mapped to regions which lie below the threshold of alternate allele frequency requirements, in general, for most bins ITD Assembler_light version is faster than using the ITD Assembler version. We observe that the resource requirements for larger bins both in terms of time and memory are negligible, and the optimization is only necessary for lower length bins. In our experience with the TCGA dataset, on samples with read length 75, all bins larger than 35 finished in less than three hours, including the preprocessing and binning steps. In conclusion, we add that while ITD Assembler_light can also detect ITD’s with extremely low variant allele coverage, ITD Assembler is also extremely sensitive in detecting reasonably strong signals (coverage >0.1) of tandem duplications or partial duplications, with limited computational resources.

## Conclusion

ITD Assembler is a highly sensitive algorithm for identification of (partial) tandem duplication events from massively parallel sequencing data. ITD Assembler is scalable to run on exome capture sequencing data, and sensitively detects partial, large and complex tandem duplications either adjoining or separated by short nucleotide distances. The presence of clinically actionable ITD mutations in AML is known; however, the extent of other such clinically relevant ITDs across cancer and Mendelian disease remains an open question, requiring execution of the ITD Assembler algorithm on additional data sets for further investigation. With the length of reads increasing each year, the limitations on the range of ITDs discovered can be easily overcome. Use of overlapping paired end reads in fragments can also be leveraged to improve upon the range while extracting signatures of tandem duplications from mate pairs where both of them are unmapped. With highly specialized detection capabilities in the range in which it can function, ITD Assembler can be combined with preexisting tools like Pindel or Genomon to get a far more comprehensive picture of the genome.

### Availability and requirements

 • **Project name:** ITD Assembler

 •**Project home page:**https://sourceforge.net/projects/itdassembler/

 • **Operating system(s):** Linux

 • **Programming language:** Haskell, Python and C.

 • **Other requirements:** Python 2.7+, SAMtools, BAMtools, Blastn, IntersectBed, PHRAP, GHC 7.8.2+

 • **License:** GNU GPL

## Additional file

Additonal file 1: Supplementary file contain ITD calling results using ITD assembler on the TCGA AML dataset. (DOCX 15145 kb)

## Abbreviations

BAM: binary alignment/map
Genomon: Genomon-ITDetector
ITD: internal tandem duplication
OLC: overlap-layout-consensus
TCGA: the cancer genome atlas
