# Supplementary material for: ITD assembler: an algorithm for internal tandem duplication discovery from short-read sequencing data
Source: BMC Bioinformatics. 2016 Apr 27;17:188. doi: 10.1186/s12859-016-1031-8 (PMC4847212; doi:10.1186/s12859-016-1031-8)
Supplement: Additonal file 1: — Supplementary file contain ITD calling results using ITD assembler on the TCGA AML dataset. (DOCX 15145 kb) [file 12859_2016_1031_MOESM1_ESM.docx]

*BMC Bioinformatics Additional file 1*

**ITD Assembler: An algorithm for internal tandem duplication discovery from short-read sequencing data**

Navin Rustagi^1*^, Oliver A Hampton^2,3*^, Jie Li^2,4^, Liu Xi^2^, Richard A. Gibbs^2,3^, Sharon E. Plon^2,3,5^, Marek Kimmel^1^, David A. Wheeler^2,3§^

*These authors have contributed equally to the paper.

§ Corresponding author

^1^Department of Statistics, Rice University, Houston TX,

^2^Human Genome Sequencing Center, Baylor College of Medicine, Houston TX,

^3^Department of Molecular and Human Genetics, Baylor College of Medicine, Houston TX,

^4^Department of Dermatology, Xiangya Hospital, Central South University, Hunan, China,
^5^Department of Pediatrics/Hematology-Oncology, Texas Children’s Hospital, Houston TX,

**ITD Discovery**

Execution of ITD Assembler on the 314 TCGA AML whole exome sequencing tumor/normal bam files resulted in identification of 1,322 somatic and 243 germline ITD mutations. ITD Assembler was executed with the following parameters: *p-kmer* = 10, *r_min* = 15 and *r_max* = 61. Somatic and germline assessment of mutations was performed using filters requiring somatic ITDs to have tumor allele fractions>0.4%, tumor variant coverage >4 reads, normal variant coverage <15 reads, and tumor allele fractions at least 10% higher than the same patients’ normal allele fraction; while, germline ITDs are required to have normal and tumor allele fractions>1.0%, normal variant coverage >10 reads, and tumor variant coverage >2 reads. Supplemental Fig. 1 shows the number of somatic and germline ITD mutation per AML patient.


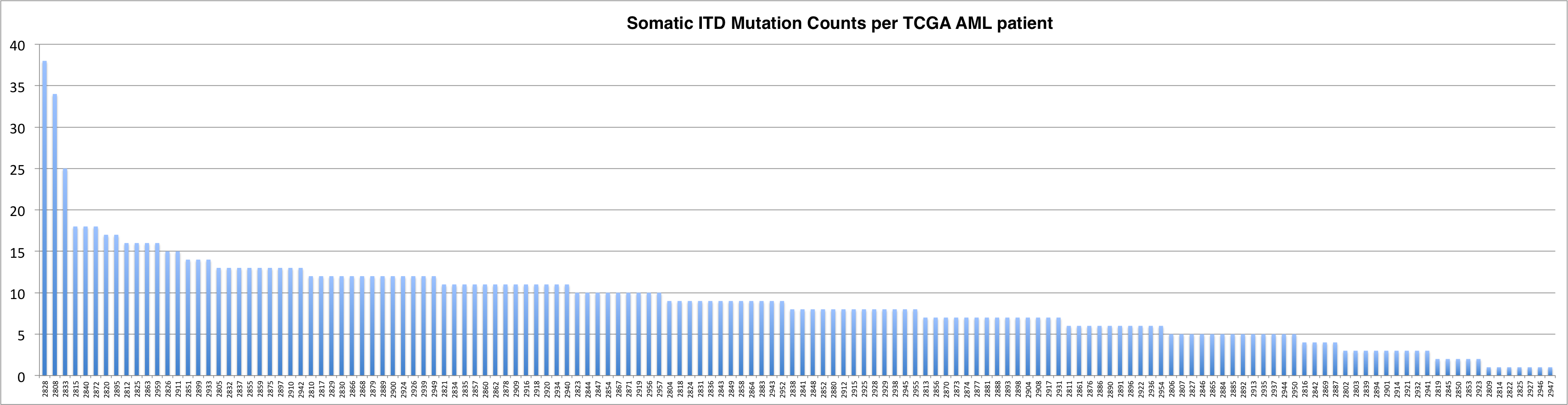


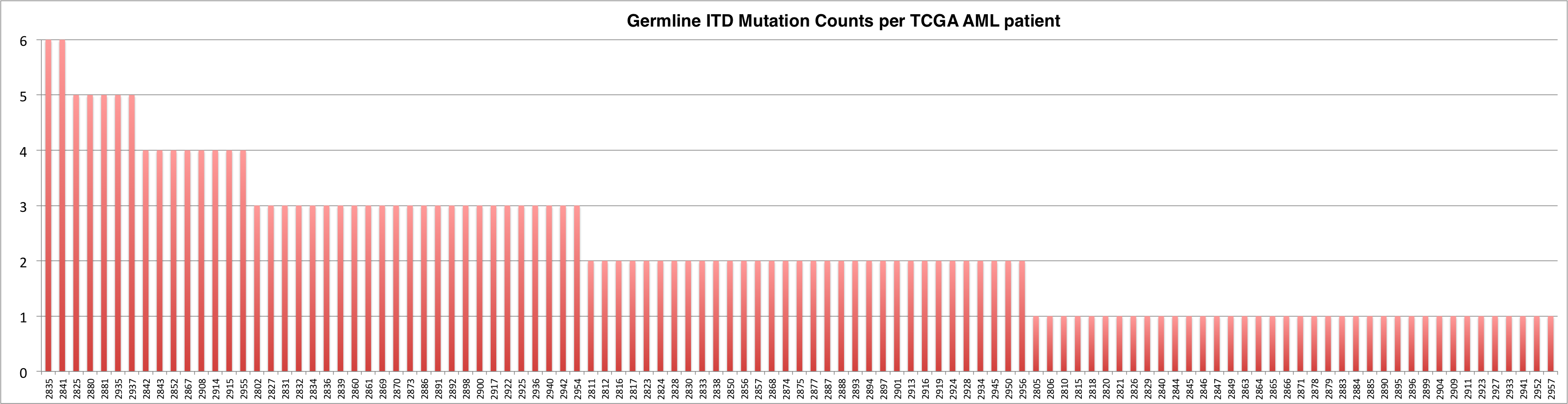


Supplemental Figure 1. ITD Assembler somatic and germline ITD mutation counts for the 157 TCGA AML patients. Somatic ITD counts are shown in blue and germline ITD counts are shown in red.

**ITD Validation**

ITDs were validated in two ways. First, by BLAST analysis of the contigs harboring each ITD. BLAST output was evaluated computationally to ensure that the assembled contig aligned to the reference with 99% identity, except for the duplicated region. Second, we used tumor sample RNAseq data that was available for 128 out of the 157 patients. On average, 153M RNAseq reads (7.65 Gb) were generated per TCGA AML tumor sample. RNAseq reads were aligned using BLAST to the assembled contigs containing putative ITDs. We required at least two RNAseq reads that align with >97% identity, and span >90% of the subject RNAseq read, and align across the ITD junction site to validate the ITD. In addition, at least ten reads align to the region surrounding the ITD insertion site. The somatic ITD validation rate was 74.3% (364 valid somatic ITDs out of 490 total tested somatic ITDs), and the germline ITD validation rate was 70.3% (45 valid germline ITDs out of 64 total tested germline ITDs).

**FLT3-ITD Contigs and Duplicated Sequences**

The somatic FLT3-ITDs reported by ITD Assembler exhibit visual evidence of good sequence quality and uniform coverage. TableView[1] visualizations of the OLC assembled contigs for the somatic FLT3-ITDs are presented below in Supplemental Figure 4, with color-coded nucleic acid contig sequence on top, followed by base pair coverage distribution plotted on the negative axis, followed by the individual assembled reads contributing to the contig with consistent color-coded nucleic acids and base mismatches highlighted in pink. Red highlighted boxes are drawn around the assembled reads and displayed above the contig indicating the duplicated sequence. (see following FLT3-ITD figures).


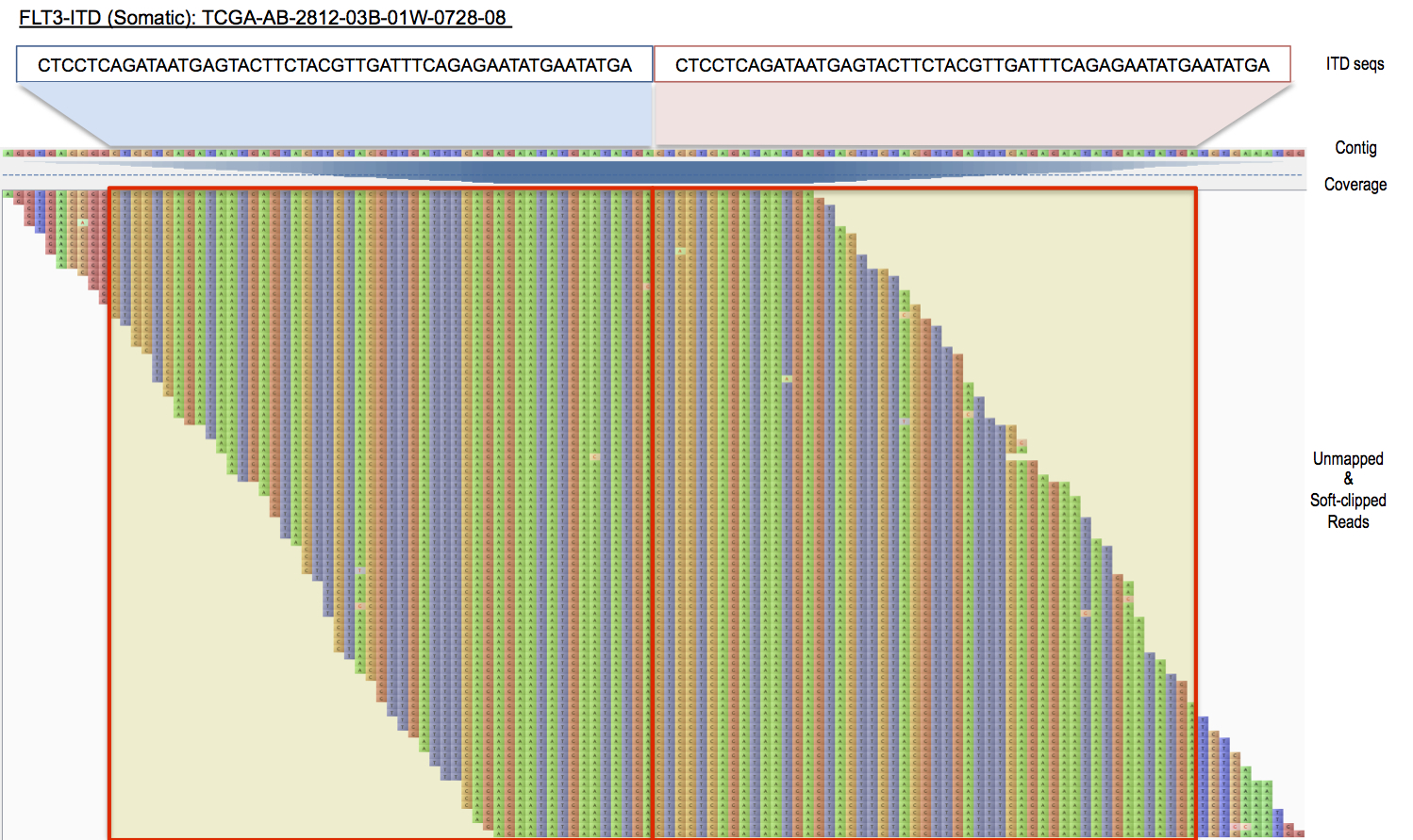


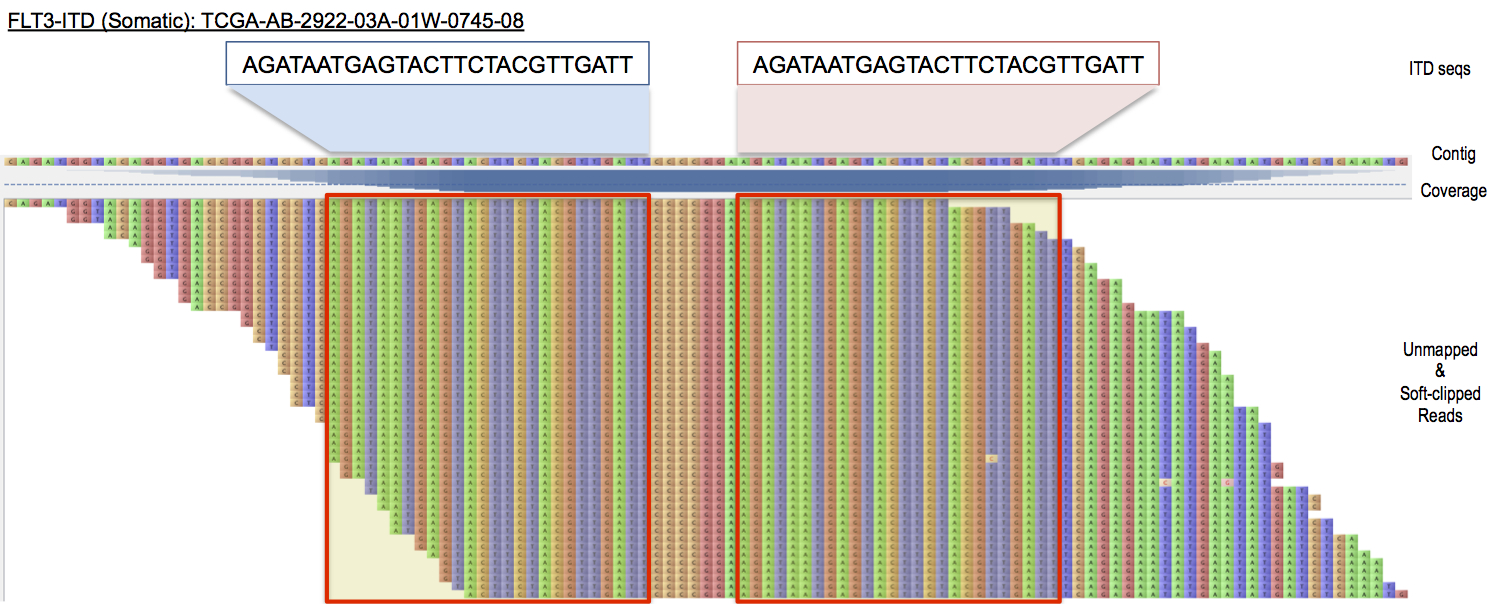


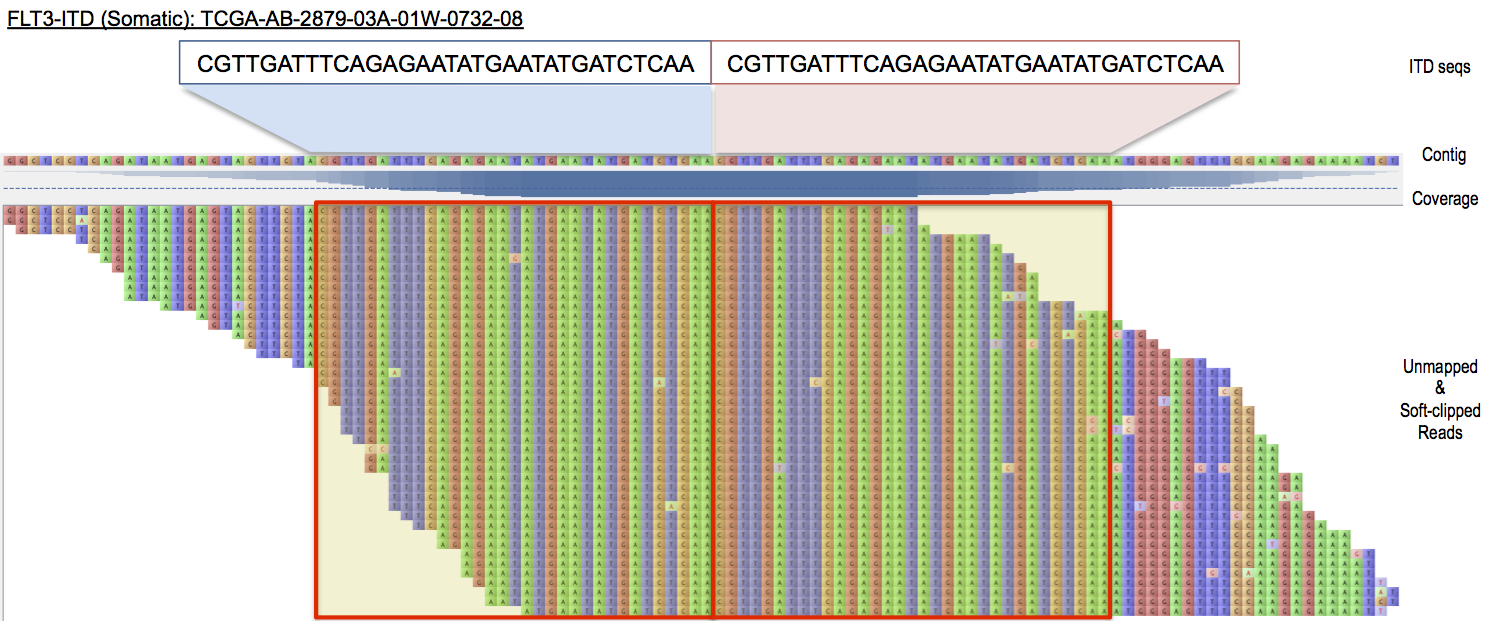


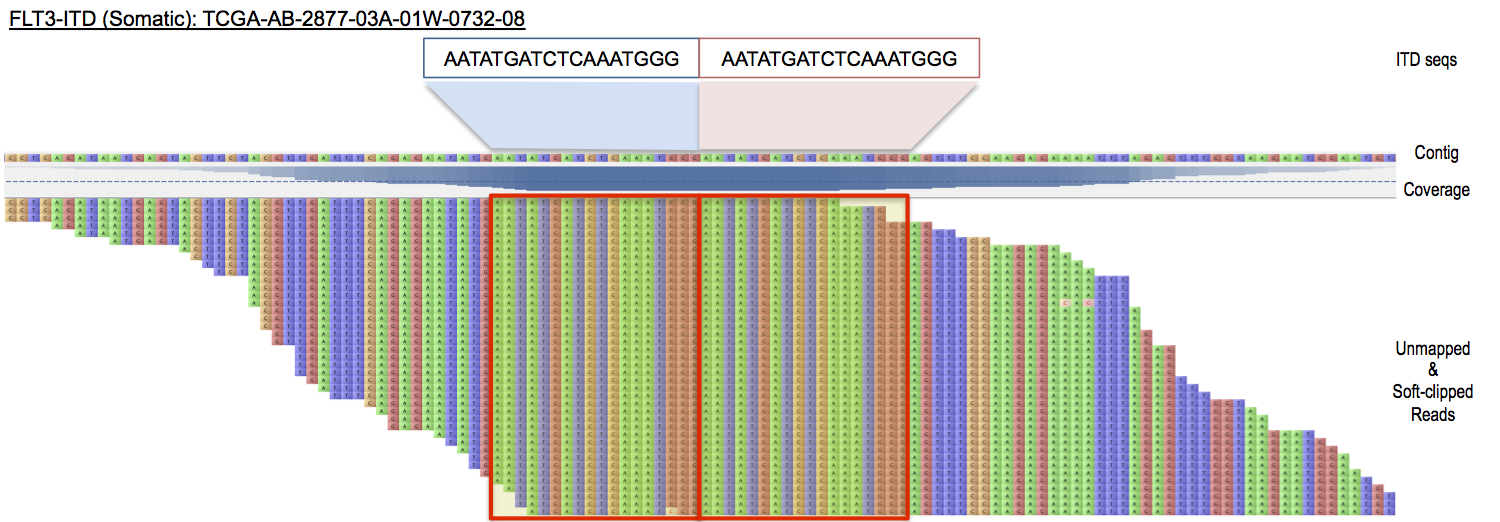


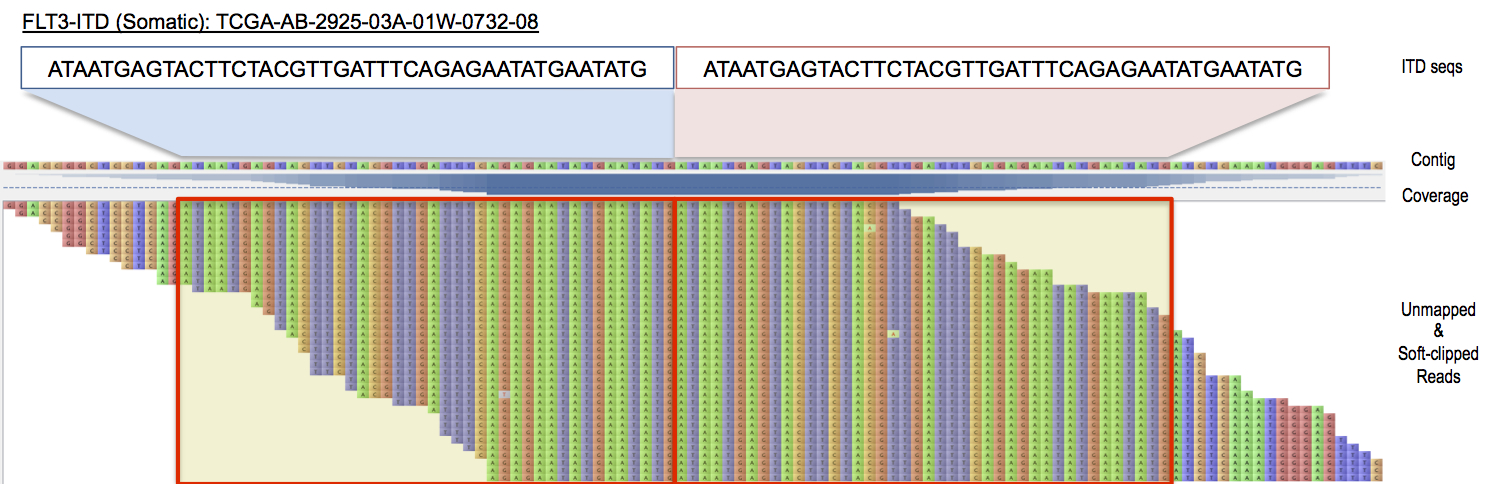


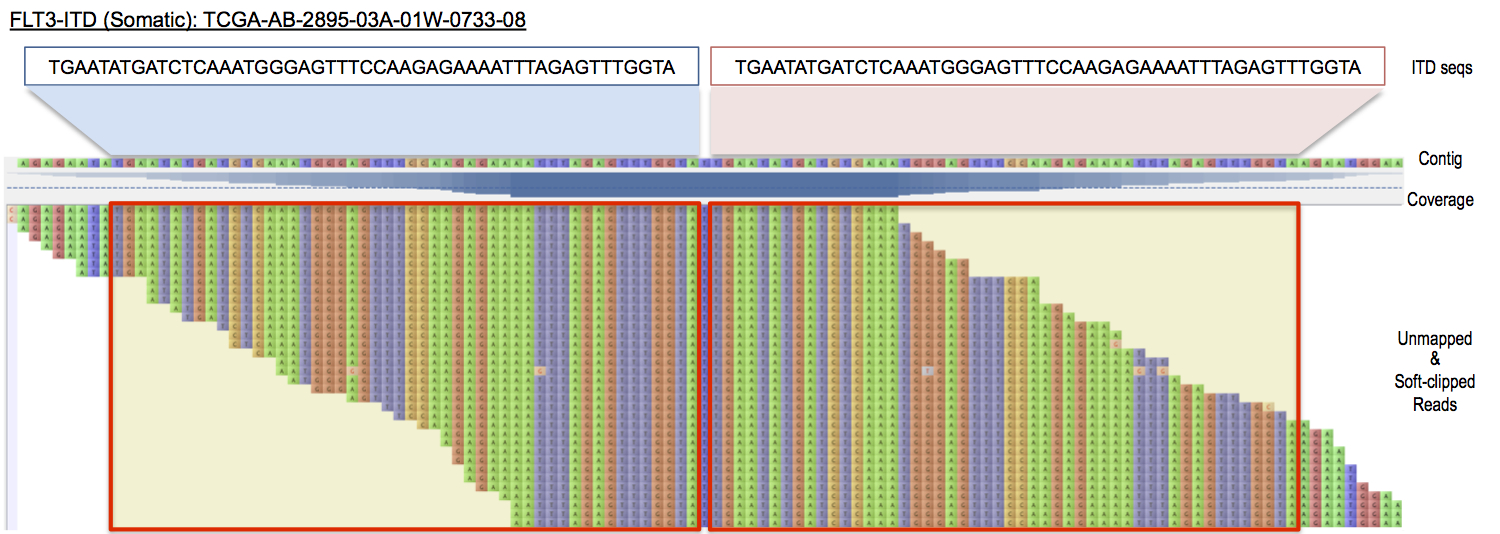


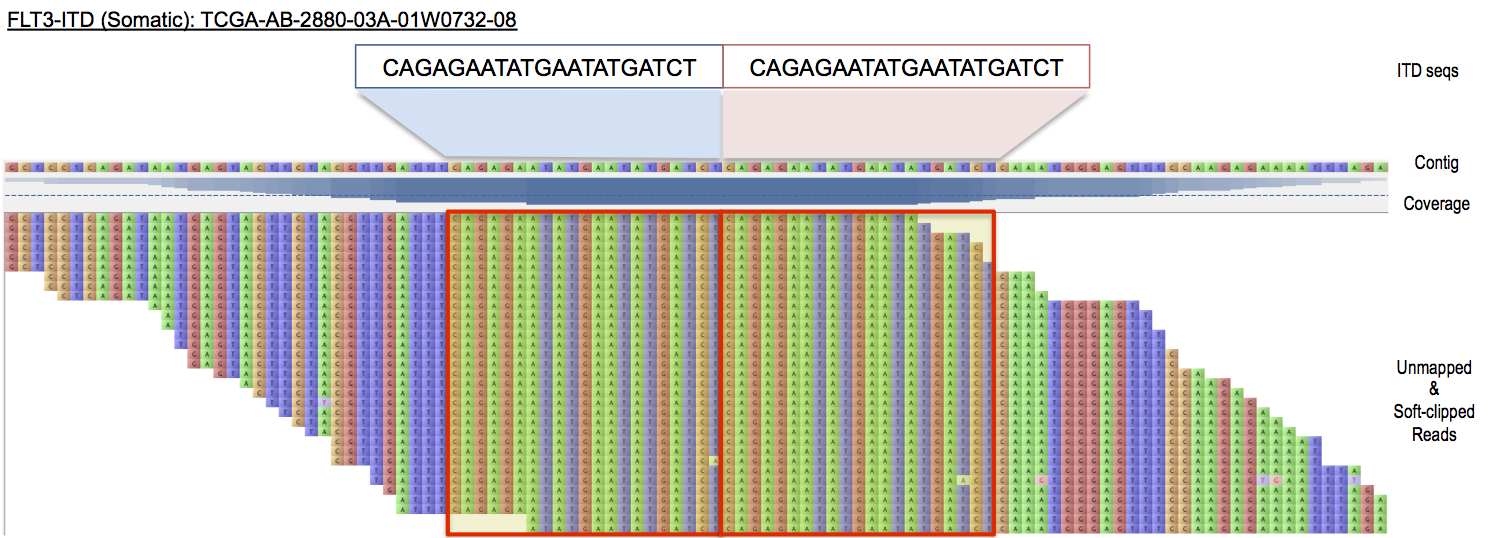


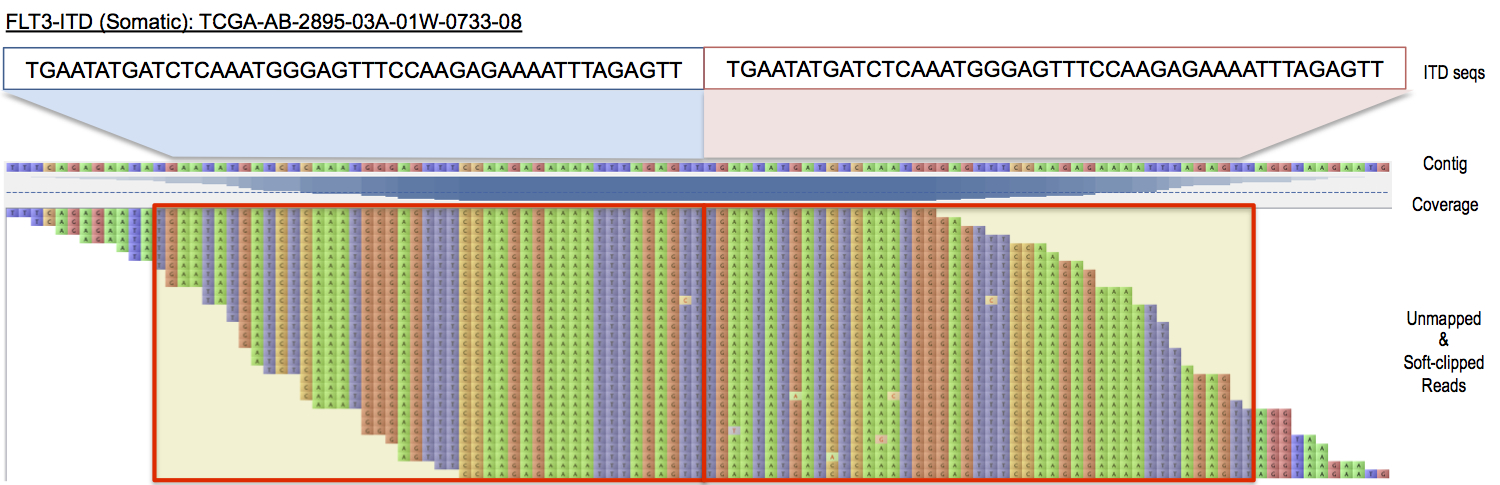


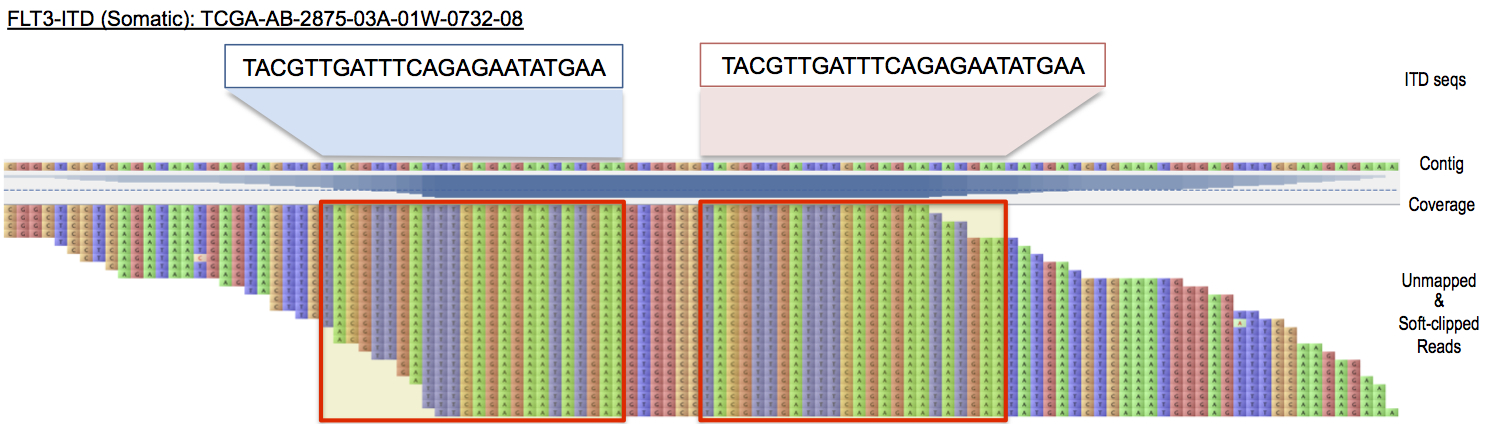


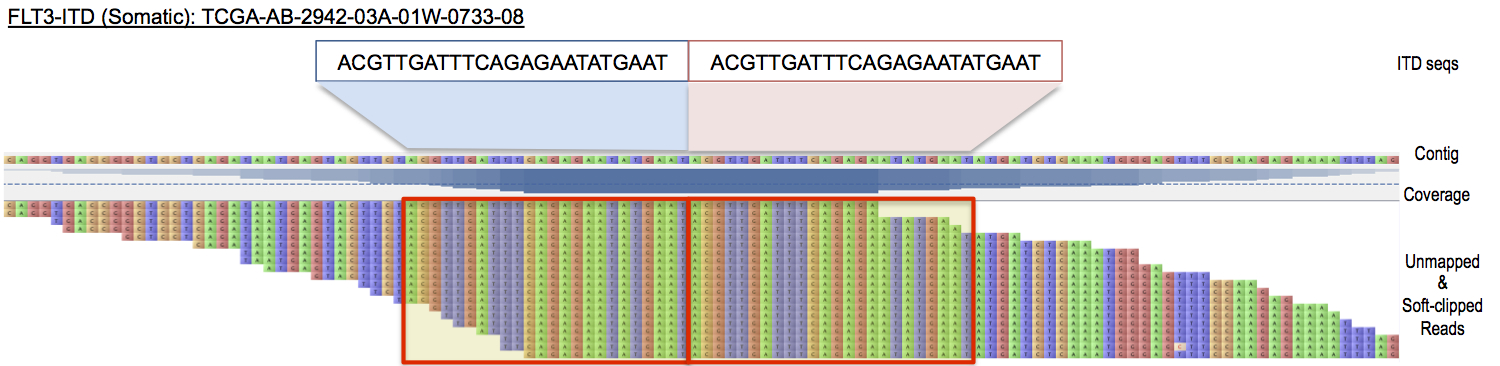


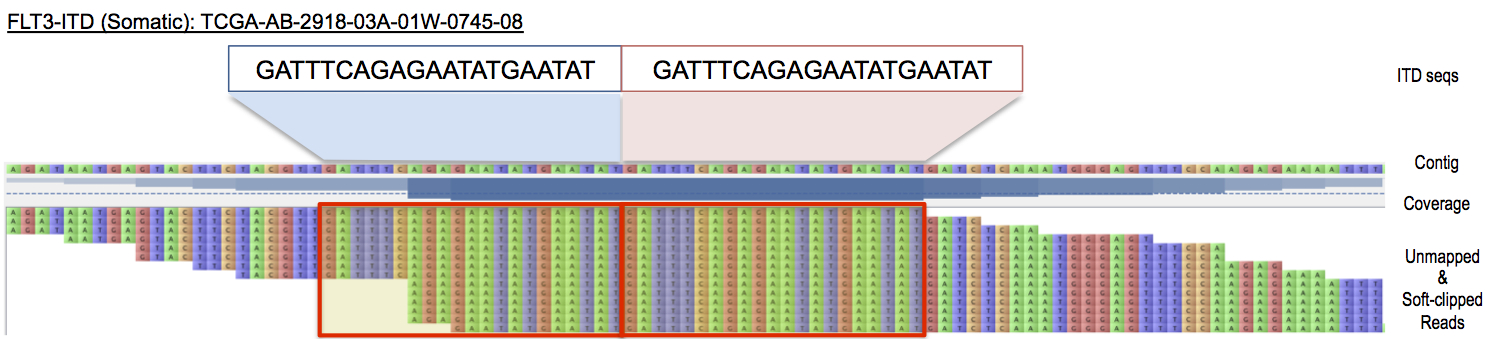


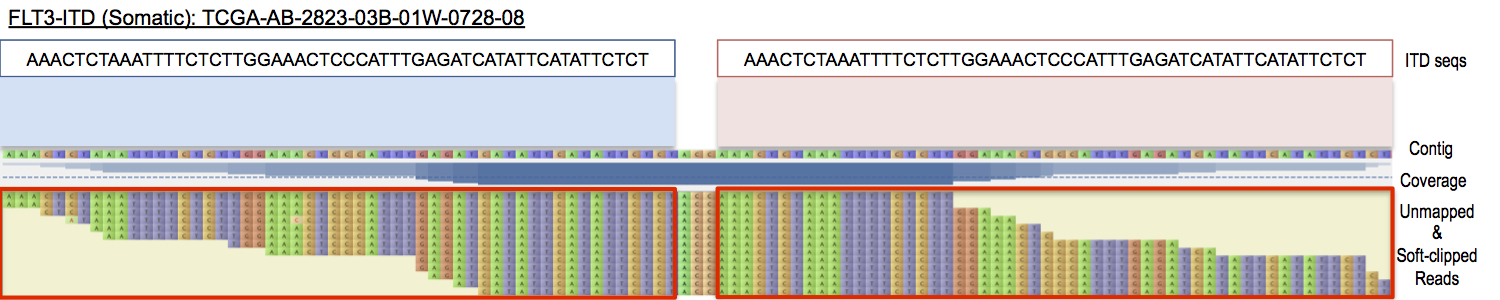


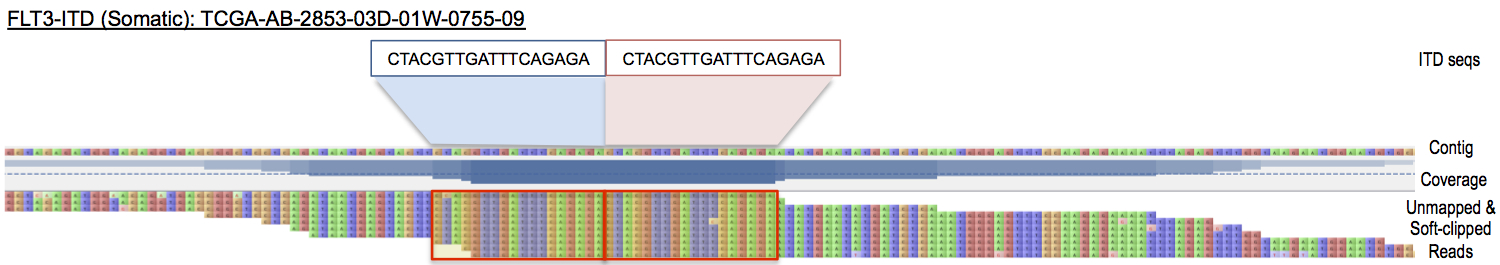


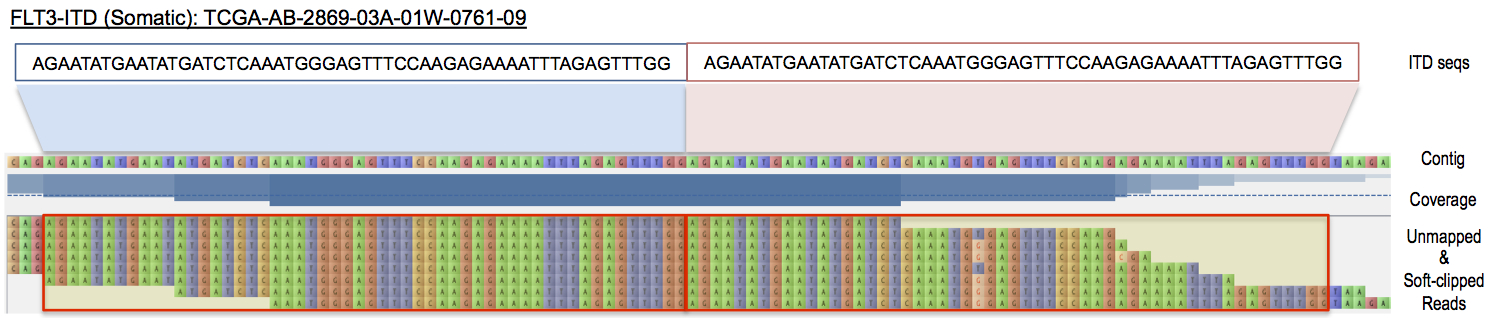


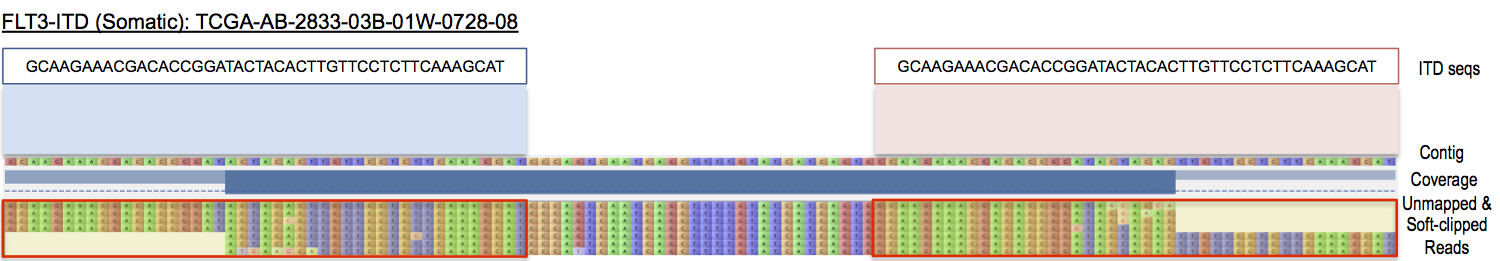


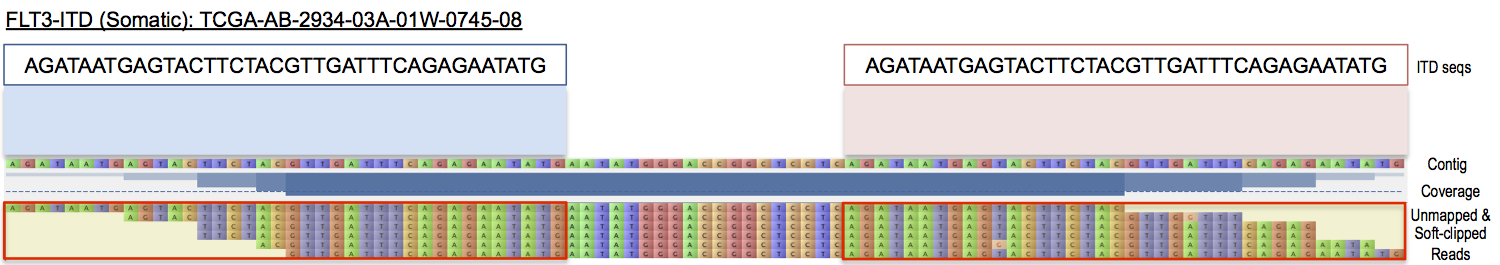


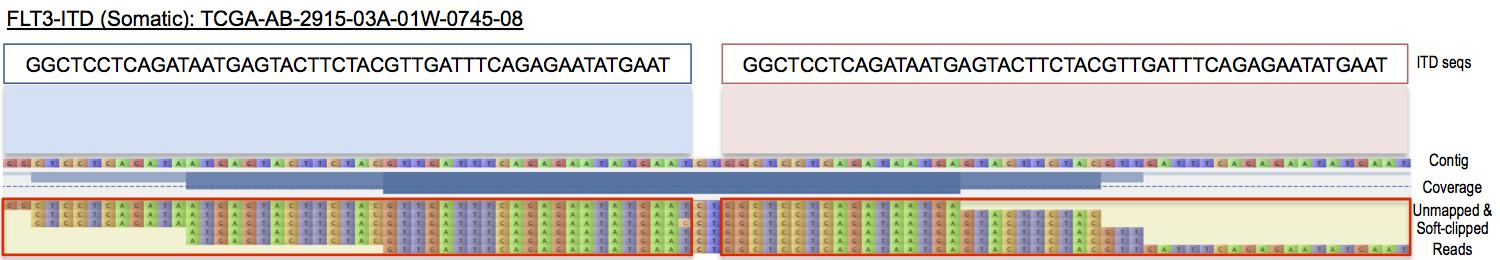


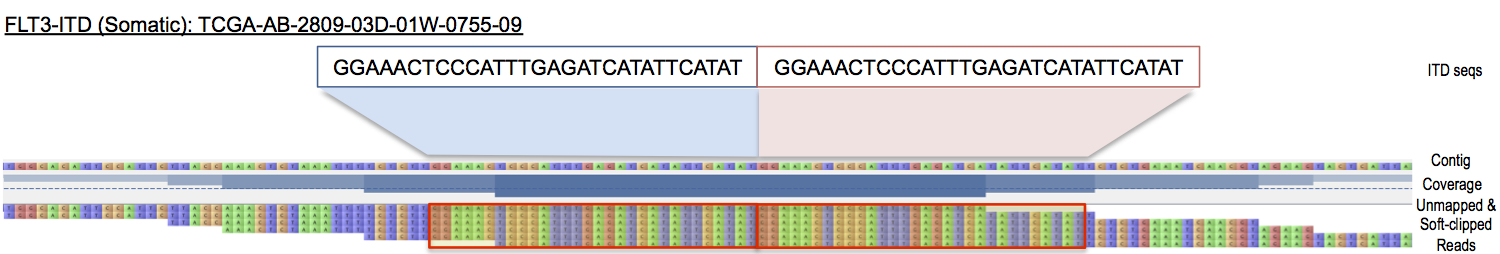


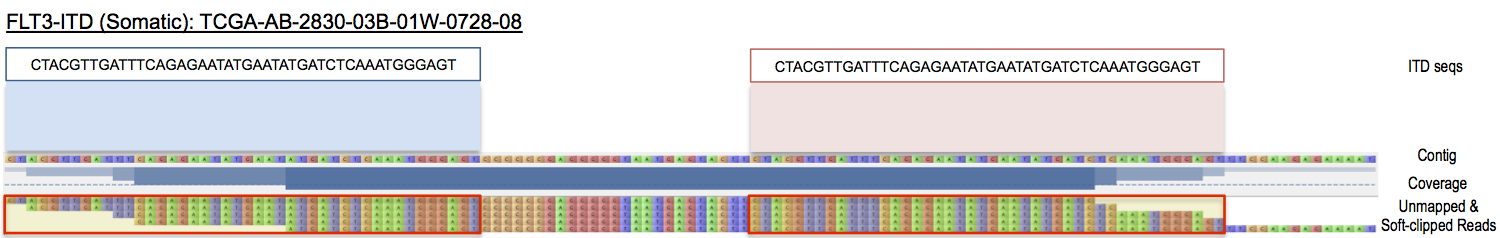


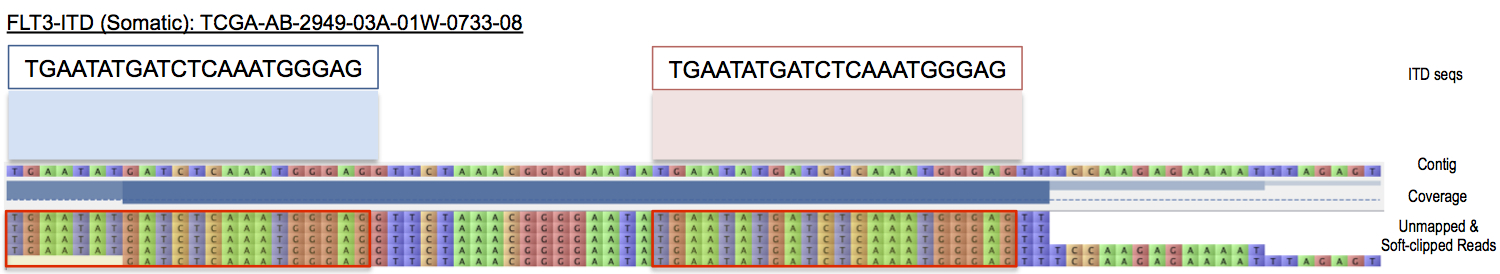


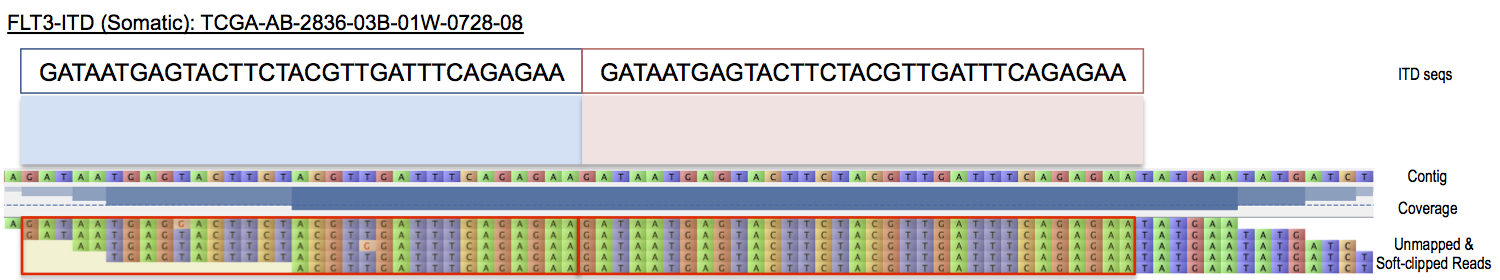


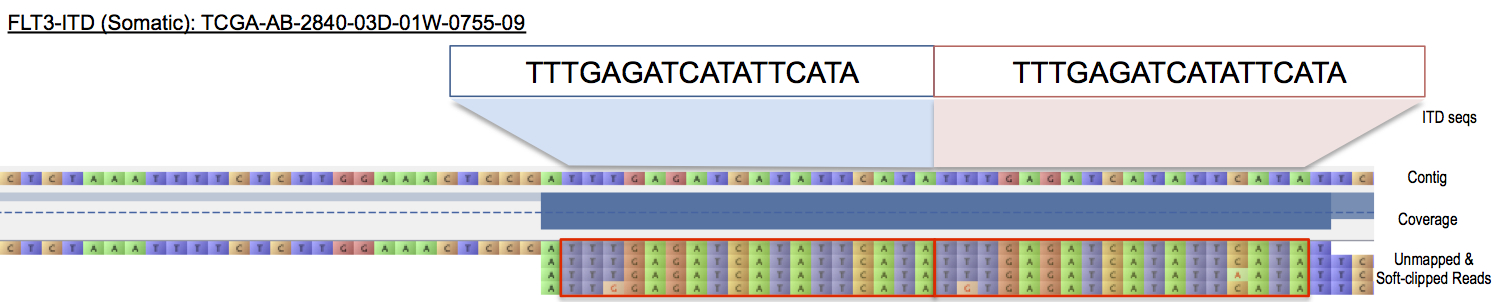


Supplemental Figure 2.TabletView[1]of the OLC assembled contigs from the somatic FLT3-ITDs reported by ITD Assembler.

**ITD Tool Comparison**

Overlap analysis of the FLT3-ITD mutations detected by ITD Assembler, Pindel, Genomon ITDetector, and TCGA Barnacle is presented below. Of the 22 FLT3-ITDs published by TCGA AML project team, ITD Assembler identified 15 (68%), while Genomon ITDetector identified 14 (64%) and Pindel identified 8 (36%). Cross-validation assessment of FLT3-ITD detection across all tools shows relatively equal sensitivity for ITD Assembler (43%), Genomon ITDetector (47%) and Barnacle v0.1.2 RNAseq analysis (50%), while Pindel exhibits less cross-validating sensitivity (28%).

Supplemental Figure 3.Venn diagram of the FLT3-ITD mutations detected in the TCGA AML patient sample cohort using ITD Assembler, Pindel, GenomonITDetector and TCGA Barnacle RNAseq analysis.

**Performance of ITDAssembler on FLT3 sensitivity**

| ID | (Light)  ITD Length | (light)  Variant AF | ITDAssembler  kmer 15 , cov_min=30 | ITDAssembler  kmer=15,cov_min=15 |
| --- | --- | --- | --- | --- |
| 2853 | 18 | 0.011 | § |  |
| 2840 | 18 | 0.011 |  |  |
| 2877 | 18 | 0.232 | § |  |
| 2880 | 21 | 0.254 | § |  |
| 2918 | 21 | 0.113 | § |  |
| 2942 | 24 | 0.159 |  | § |
| 2875 | 30 | 0.187 | § |  |
| 2836 | 33 | 0.054 |  |  |
| 2879 | 33 | 0.355 | § |  |
| 2922 | 33 | 0.262 | § |  |
| 2925 | 42 | 0.291 | § |  |
| 2895 | 45 | 0.201 | § |  |
| 2812 | 51 | 0.412 | § |  |
| 2869 | 54 | 0.017 | § |  |
| 2830 | 69 | 0.038 |  |  |
| 2809 | 30 | 0.019 | § |  |
| 2949 | 39 | 0.021 | § |  |
| 2915 | 51 | 0.058 | § |  |
| 2895 | 51 | 0.228 | § |  |
| 2934 | 57 | 0.058 |  |  |
| 2823 | 57 | 0.137 |  | § |
| 2833 | 75 | 0.004 |  |  |

Table S1 : Results of detectection sensitivity of ITDAssembler on various cutoffs for ITDs which are detected by ITDAssembler light.


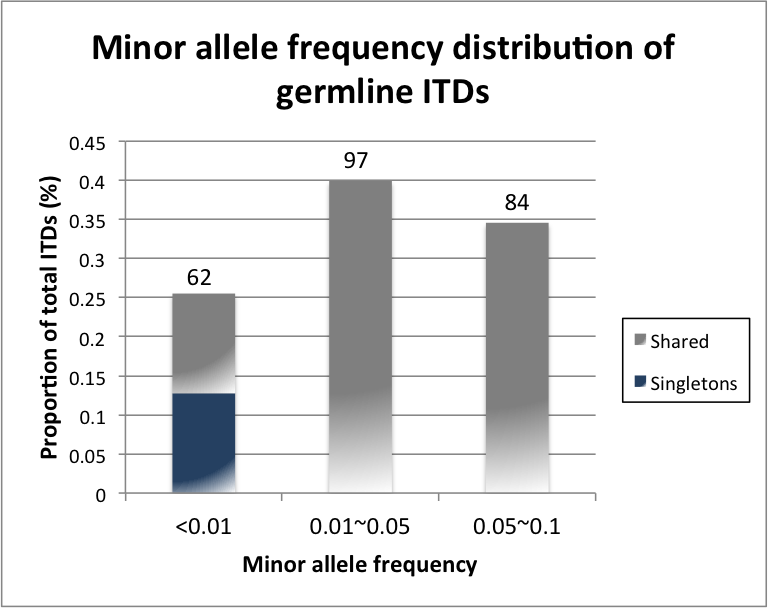


Supplemental Figure 4: Minor Allele Frequency Distribution of the 243 germline ITD’s. The majority of germline ITDs are shared among the LAML patient population with only 31 singleton germline ITDs being called by ITD-ASM.

**References**

[1] Milne, I. *et al.* Using Tablet for visual exploration of second-generation sequencing data. *Brief. Bioinform.***14,** 193–202 (2013).
